# Contrast echocardiography in daily clinical practice

**DOI:** 10.1007/s00059-017-4533-x

**Published:** 2017-02-03

**Authors:** M. Eskandari, M. Monaghan

**Affiliations:** King’s College Hospital, London, UK

**Keywords:** Perfusion imaging, Ultrasonography, Contrast media, Coronary artery disease, Left ventricle, Perfusionsbildgebung, Sonographie, Kontrastmittel, Koronare Herzkrankheit, Linker Ventrikel

## Abstract

Ultrasound contrast agents have unique acoustic properties that enable them to enhance the cardiac blood flow and thus are used broadly in modern echocardiography laboratories for salvage of nondiagnostic studies, improving accuracy and reducing variability even in the presence of adequate image quality. Contrast echocardiography is also used as an adjunctive technique when unenhanced echocardiography falls short in the differentiation of cardiac structural abnormalities such as cardiac masses. Ultrasound contrast agents are pure intravascular tracers. Development of innovative ultrasound imaging techniques has led to myocardial perfusion imaging with contrast echocardiography. Although currently an off-label indication, it has been shown that perfusion imaging with contrast echocardiography adds incremental value to stress echocardiography in the detection of coronary artery disease. Moreover, it can be used for assessment of myocardial viability. In this paper we briefly discuss the basics of contrast echocardiography and its use in daily clinical practice.

## Zusammenfassung

Ultraschallkontrastmittel weisen besondere akustische Eigenschaften auf, durch die der kardiale Blutfluss verstärkt dargestellt wird. Daher ist ihre Anwendung in der modernen Echokardiographie weit verbreitet, um trotz einer wenig aussagekräftigen Untersuchung noch zu einem Ergebnis zu kommen, die Genauigkeit zu erhöhen und die Variabilität selbst bei ausreichender Bildqualität zu senken. Die Kontrastechokardiographie dient auch als Zusatzverfahren, wenn die native Echokardiographie zur Differenzierung kardialer Strukturanomalien wie kardiale Raumforderungen nicht ausreicht. Ultraschallkontrastmittel sind rein intravaskuläre Tracer. Die Entwicklung innovativer bildgebender Verfahren hat zur Myokardperfusionsbildgebung mit Kontrastechokardiographie geführt. Obwohl es sich derzeit um eine Off-Label-Indikation handelt, weist die Perfusionsbildgebung mit Kontrastechokardiographie nachweislich einen zusätzlichen Nutzen zur Stressechokardiographie bei der Erkennung der koronaren Herzkrankheit auf. Darüber hinaus kann das Verfahren zur Beurteilung der Erholungsfähigkeit von kontraktionsgestörtem Myokard eingesetzt werden. In der vorliegenden Arbeit werden die Grundlagen der Kontrastechokardiographie und ihre Anwendung in der täglichen klinischen Praxis kurz erörtert.

Despite technological advances in echocardiography, such as harmonic imaging, a significant number of rest or stress echocardiography studies are labeled as nondiagnostic. Even in the presence of adequate image quality, some cardiac structural abnormalities pose a diagnostic challenge. Ultrasound contrast agents (UCAs) have been shown to improve the diagnostic accuracy of echocardiography. Furthermore, assessment of myocardial perfusion by means of contrast echocardiography has been extensively studied with proven independent diagnostic and prognostic benefits. This paper (a) briefly highlights some technical aspects of UCAs and the relevant imaging techniques and (b) summarizes the use of contrast echocardiography in daily clinical practice.

## Ultrasound contrast agents

Use of UCAs in echocardiography is not new. In 1968, Gramiac and Shah noted a cloud of echo at the tip of cardiac catheters while doing M‑mode studies on the aortic root in the cardiac catheterization laboratory [1]. Injection of agitated saline for identifying right-to-left intracardiac shunts or pulmonary arteriovenous malformation continues to remain a valuable clinical tool in daily practice. Air microbubbles that act as the contrast agent are approximately the same size as red blood cells with strong backscatter; however, they dissolve in blood quickly and therefore do not normally reach the left heart. Despite knowing for many years that mixing a small amount of blood with agitated saline increases microbubble stability (as blood surfactant forms a protective shell around the microbubble), it was only in early 1990s that commercial UCAs became available [2]. Thus, in order to overcome the instability of contrast microbubbles in blood, they should be either protected with a shell or should contain a gas that is insoluble in the blood so that they can reach the left ventricular cavity and the myocardium. The first generation of UCAs had air or nitrogen microbubbles protected by a thick shell (such as albumin). The second generation of UCAs instead has high-molecular-weight gases that are insoluble in blood. UCAs should also ideally have distinct acoustic properties to provide efficient backscatter, have a similar size to red cells in the blood, and circulation and must survive the pulmonary passage without aggregation. The most important factor in the echogenicity of the UCAs is their size, and therefore the biggest possible size of a UCA that is able to cross the lungs will produce the highest ultrasound backscatter. Table 1 summarizes some of the commercially available UCAs. Unlike red cells that only become echogenic in aggregation and slow blood flow state, UCAs produce ultrasound backscatter even in normal blood flow that is typically up to 100 million times stronger than red cells.

**Table 1:** Commercially available UCAs [2, 6]

Agent	Bubble size (μ)	Gas	Shell	Approved
Optison	3–4.5	Perfluorocarbon (C3F8)	Human albumin	EU, USA
Sono Vue (Lumason in USA)	2–8	Sulfur hexafluoride (SF6)	Phospholipids	Europe, USA, China, South Korea, India, Hong Kong, Singapore
Definity (Luminity in EU)	1.1–2.5	Perfluorocarbon (C3F8)	Phospholipids	Worldwide
Sonazoid	3	Perfluorobutane	Phospholipids	Japan

The acoustic behavior of UCAs in response to the different range in ultrasound mechanical index (MI) is an important part of contrast echocardiography. MI is directly proportional to an ultrasound beam’s peak negative pressure and inversely proportional to the frequency of the beam and is considered as the ultrasound acoustic power. In an MI > 0.1, contrast microbubbles contract and expand unequally, which means they will produce ultrasound backscatter with a lower amplitude than the transmitted fundamental frequency, the so-called nonlinear response. In lower MI myocardial tissue backscatter, however, typically shows a linear response, i. e., producing backscatter signals at the same amplitude. Modern scanners will be able to cancel out the tissue backscatter and image only reflected signals from the UCA. An MI of more than 0.9 will cause destruction of the UCA resulting in brief but strong nonlinear backscatter signals that also can be used in contrast echocardiography. In summary, the two techniques are called low-MI and high-MI imaging, respectively. The real-time low-MI (<0.3) techniques are those that are more routinely used in clinical practice [3].

## Clinical utility of contrast echocardiography

Contrast echocardiography is used for a variety of diagnostic and prognostic reasons. Table 2 summarizes the clinical indications of contrast echocardiography recommended by major society guidelines.

**Table 2:** Clinical indications for contrast echocardiography

1. Quantification of left ventricular (LV) volumes and LV ejection fraction, assessment of regional wall motion abnormality at rest – Improvement in endocardial border delineation in difficult-to-image patients – To increase accuracy and reproducibility in all patients – To increase reader confidence in all patients
2. LV structure abnormalities – Apical LV thrombus – Apical hypertrophic cardiomyopathy – LV Non-compaction – LV rupture, aneurysm, and pseudo-aneurysm 2. Cardiac masses, thrombus
3. Improvement in right ventricle visualization and great vessels 3. Enhancement of Doppler signals for evaluation of valvular disease/diastolic function
4. Stress echocardiography
5. Perfusion imaging with myocardial contrast echocardiography

### Left ventricular opacification

#### Left ventricular function

Assessment of global and regional left ventricular function has both diagnostic and prognostic value in a range of cardiac conditions such as coronary artery disease, heart failure, and cardio-oncology. It is well reported that up to 15% of echocardiography studies are uninterpretable owing to poor image quality. This is even more prominent in critically ill patients and has been reported to be as high as 30% [4]. Both American and European guidelines advocate the use of UCAs to improve endocardial border delineation when two or more segments of the left ventricle cannot be well defined [5, 6]. The salvage of such nondiagnostic studies using left ventricular opacification (LVO) has been reported to be as high as approximately 50% with higher rates of salvage in the intensive care unit [7–9]. It is only with the advent of contrast echocardiography that the percentage of nondiagnostic echocardiography studies has dropped to less than 5% [4]. Moreover, quantification of ejection fraction is imperative in daily practice both in clinical decision-making, e.g., eligibility for device therapy with an ejection fraction <35%, and in serial studies such as monitoring of the left ventricular function in valvular heart disease. Measurement of ejection fraction by unenhanced two-dimensional (2D) echocardiography has inherent limitations resulting in underestimation by up to 6% and a significant variability of up to 14% [10, 11]. Several studies have shown that LVO improves the accuracy of ejection fraction measurements and reproducibility [6] and is recommended in relevant clinical indications. UCAs have also been used with three-dimensional (3D) echocardiography. Recent studies have shown that 3D contrast echocardiography provides the closest results to cardiac magnetic resonance in measuring left ventricular volumes. It further reduces variability and improves accuracy of left ventricle volume measurements when compared with 2D contrast echocardiography [12, 13].

##### Image Optimization.

**Fig. 1:**
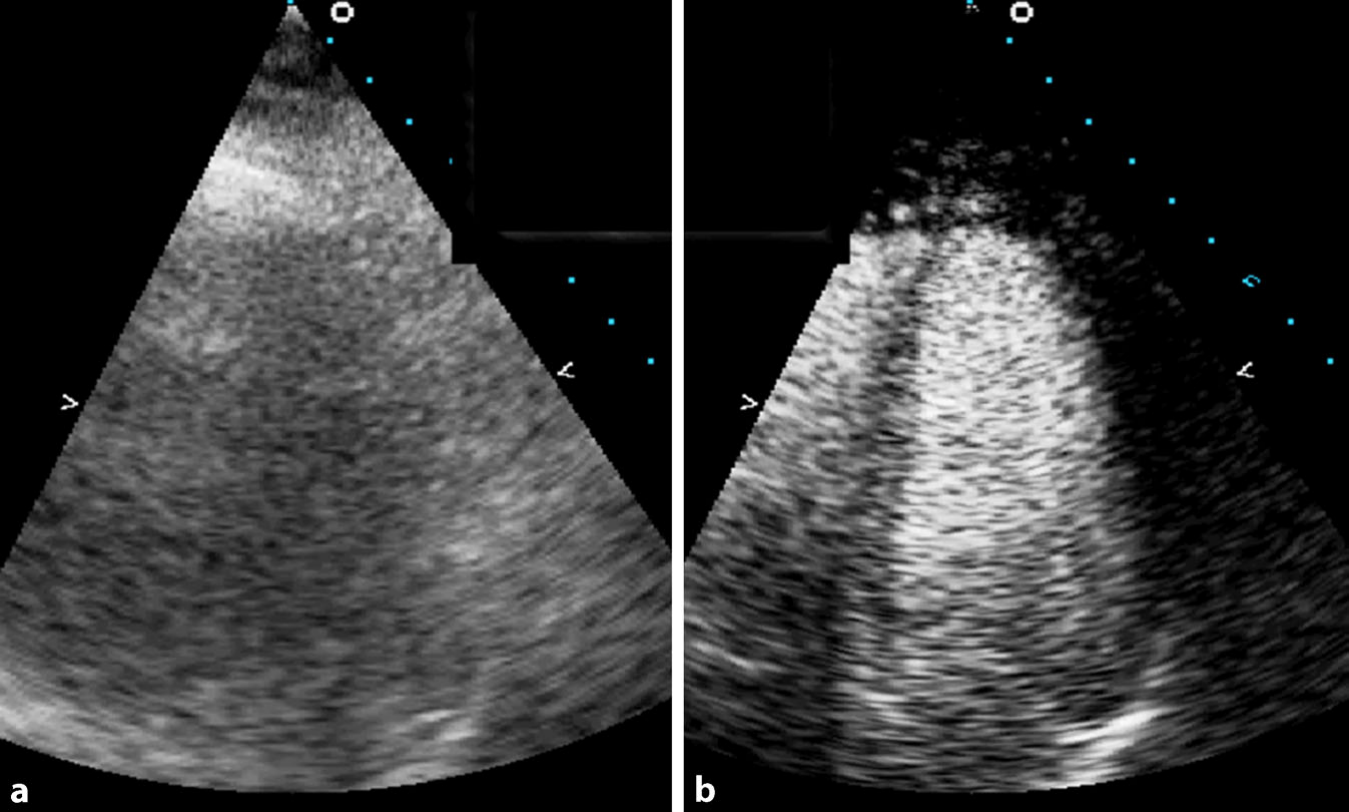
**a** A nondiagnostic four-chamber apical view obtained from a patient with severe chronic obstructive pulmonary disease. **b** Performing a left ventricular opacification study improved the endocardial definition

Conventional 2D echocardiography uses high MI that results in destruction of contrast microbubbles. When performing an LVO study, this will result in contrast swirling, especially in the apical area that is closer to the ultrasound beam source. Furthermore, at high MI, tissue will also produce harmonic backscatters (nonlinear response) that make delineation of the endocardial border more difficult. For these reasons, LVO studies should be performed with low MI (<0.3), which is usually available on modern echocardiography machines, to reduce microbubble destruction and tissue signals. The focal zone should be at the level of the mitral valve annulus and a minimum frame rate of 25 MHz is required. Adjusting time gain compensation helps to achieve a homogeneous opacification of the left ventricle from the apex to the base. Bolus injections are usually used for LVO studies and should be of such volume that adequate opacification of the left ventricle is reached. In high volumes of contrast, however, microbubbles can act as a barrier to the ultrasound beam and cause attenuation resulting in the far field view being attenuated. This can be resolved by using the “flash” or “burst” function that sends high-MI ultrasound beams over 4–5 frames and destroys the contrast allowing for a better differentiation of the endocardial border (Fig. 1).

### Cardiac structural abnormalities

#### Intracardiac masses

**Fig. 2:**
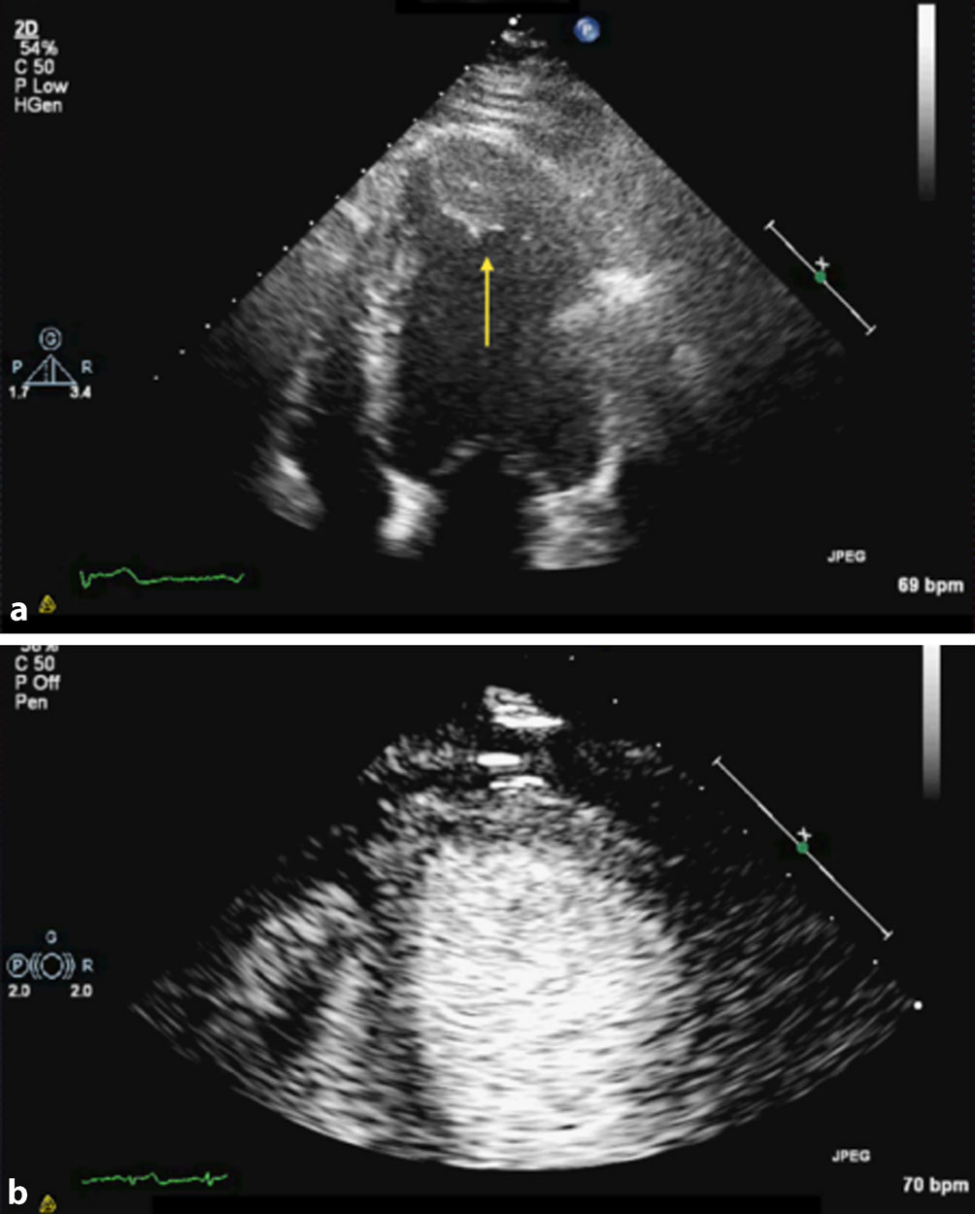
**a, b** A left ventricular opacification study that excludes suspected apical thrombus (*yellow arrow*) in the left ventricle

**Fig. 3:**
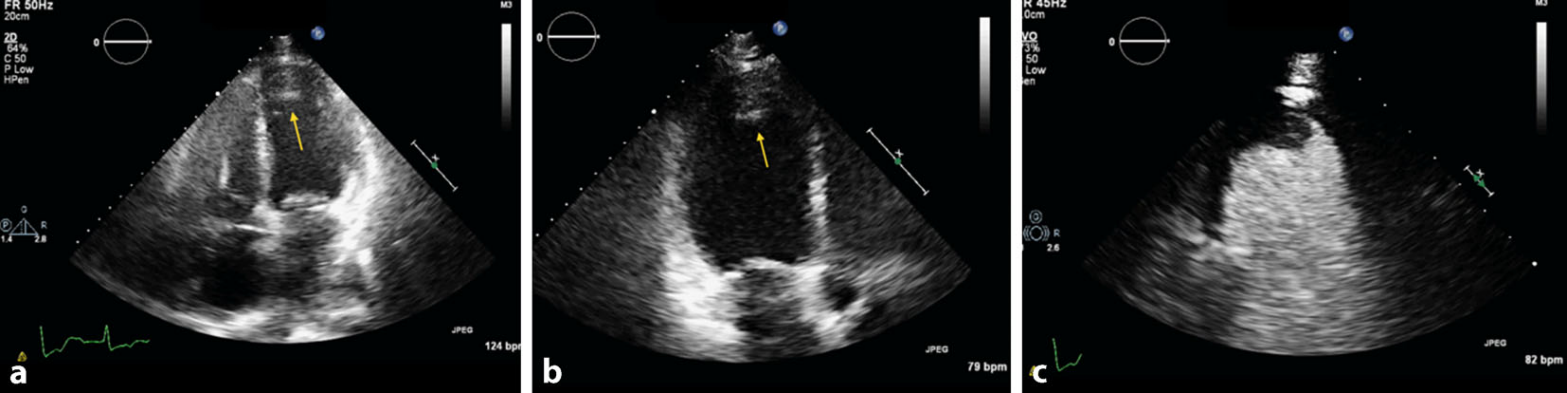
**a–c** Confirmation of apical thrombus in the left ventricle apex in a left ventricular opacification study showing a filling defect (*yellow arrow*)

Transthoracic echocardiography has a sensitivity as high as 93% for diagnosing cardiac masses [14]. Left ventricular thrombus is the most commonly diagnosed mass that can pose a diagnostic challenge, especially in cases of poor image quality or near-field artifact. Use of real-time low-MI LVO will help delineate a mural or laminar thrombus (Figs. 2 and 3). Using the flash option that destroys the microbubbles allows for visual assessment of replenishment of the adjacent myocardium to determine any perfusion defects, implying a substrate for thrombus formation. Although in the majority of cases other diagnostic clues will help in differentiating thrombus from a tumor, destruction/reperfusion techniques can be used to characterize the mass vascularity. A thrombus will be avascular, whereas a malignant mass or vascularized tumor will show high vascularity, and a stromal mass will present as partially vascular.

Diagnosis of suspected *apical hypertrophic cardiomyopathy* on transthoracic echocardiography can be confirmed by an LVO study demonstrating the spade-shape apex with the thick apical cap and apical systolic cavity obliteration. Contrast LVO can also help differentiate prominent trabecula from noncompaction morphology.

LVO can also be used in the detection of acute myocardial infarction complications such as pseudo-aneurysm. It has also been used with transesophageal echocardiography to facilitate the diagnosis of left atrial appendage closure particularly before direct-current cardioversion [15].

### Stress echocardiography

The aforementioned limitations of transthoracic echocardiography in visualization of the endocardial border can pose an even more significant challenge in stress echocardiography, when definition of the endocardial border for detection of regional wall motion abnormalities is crucial. Even in cases with adequate image quality, the respiratory effort in the case of an exercise test or tachycardia resulting from use of inotropic agents and the narrow window in acquiring images could all lead to difficulty in interpreting the studies. Suboptimal studies have been reported as high as 30% in routine stress echocardiography with an inter-institutional agreement as low as 43% in those with poor image quality. LVO has been shown to reduce the nondiagnostic stress echo studies to less than 10%, to improve test accuracy and reader confidence, and to reduce variability in several studies [16–21].

Again, real-time low-MI imaging is the preferred method. While bolus injections are easier to adopt for exercise stress echocardiography and should be administered about 30 s before acquiring images, an infusion can be used in pharmacologic stress echocardiography. Given that the infusion of the contrast takes longer to reach the adequate concentration, it is best started at least 45 s before image acquisition is performed. As discussed, a higher concentration of microbubbles can act as a barrier to the ultrasound beam causing attenuation. Thus stress echo imaging should be started with apical views since attenuation from the higher concentration of the microbubbles in the right ventricle can affect the image quality of the parasternal left ventricular views.

LVO for improvement of endocardial border definition both at rest and stress along with its role in defining cardiac structural abnormalities are the licensed applications for the use of UCAs and should be an integral part of any echocardiography laboratory. Contrast echocardiography can also be used for enhancement of Doppler signals. This can be achieved even after fragmentation of contrast microbubbles, in the absence of visible opacification of the cardiac cavities, owing to remaining microbubble shells in the circulation. More advanced use of UCAs includes myocardial contrast echocardiography (MCE), which is technically challenging and requires significant experience. The adoption of this technique, despite several favorable clinical studies, has been slow and is limited to expert centers.

### Myocardial contrast echocardiography

Coronary blood flow is equally distributed between the arteries and arterioles, capillary network, and venules. Myocardial blood volume represents a third of total coronary flow, of which more than 90% resides in the capillary network [22]. MCE relies on the intensity of the backscattered ultrasound from the filled capillaries with UCA and suppression of those reflected from the myocardial tissue. UCAs are pure intravascular traces and if they are the only source of the backscattered signals then the intensity of such signals will be proportionate to the myocardial blood volume. Providing the rate of myocardial blood filling by the contrast microbubbles is measurable, then the myocardial blood flow, which is a product of myocardial blood volume multiplied by velocity, can be calculated.

There are essentially two main MCE imaging techniques.

*Destructive (high MI) imaging techniques *use an MI > 1 that will cause microbubble destruction, resulting in a nonlinear response and generation of strong harmonic signals in multiples of the transmitted fundamental frequency. However, after the destruction phase, the myocardium will require some time to be refilled with contrast. Therefore, it is not possible to perform real-time MCE and imaging should be done by triggering ultrasound every few frames (1, 2, 3, and up to10). This means that assessment of wall motion abnormality with triggered imaging is not possible, which is considered an important disadvantage. Moreover, it is difficult to maintain the same imaging window in-between triggered frames. For these reasons, high-MI MCE is not the preferred modality in daily practice.

The two main destructive techniques are* harmonic power Doppler *and* pulse inversion Doppler. *In *harmonic power Doppler,* two consecutive pulses are transmitted in the same scan line. The reflected backscatter from the first pulse will be from both tissue and myocardial capillaries, while the signals from the second pulse will be solely from myocardial tissue (because of microbubble destruction by the first pulse). If the myocardial capillaries are not filled with contrast because of a perfusion defect, then there will not be a shift in frequency, whereas a normally perfused myocardium will result in backscatter signals from both tissue and microbubbles from the first pulse and only the tissue from the second pulse. In *pulse inversion Doppler,* two pulses are sent down shortly after each other that are 180° out of phase. Since the response of the myocardial tissue to the transmitted signal will be linear, they will cancel each other out. However, microbubbles will respond in a nonlinear fashion and therefore the backscattered signal intensity will be a reflection of the contrast microbubbles in the myocardial capillaries. At high MI, myocardial tissue will also produce harmonics that will be filtered out using modern narrow-band transducers.

**Fig. 4:**
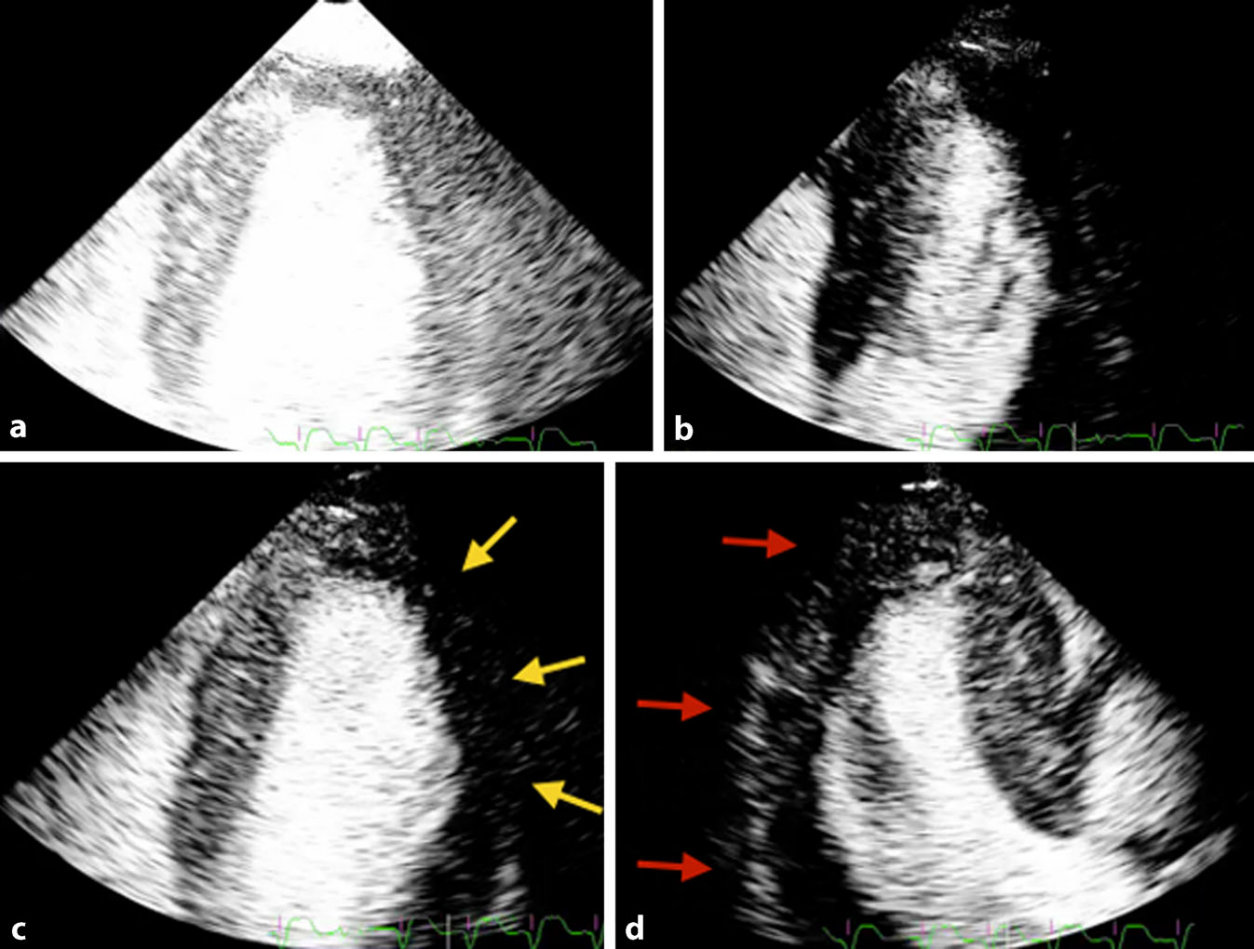
Real-time low-MI myocardial contrast echocardiography. **a,** **b** Immediately after flash function; **c** as contrast appears in the myocardium, *yellow arrows* show a large area of perfusion defect in the lateral wall in the apical four-chamber view; **d** *red arrows* show the perfusion defect in the inferolateral wall in the apical three-chamber view. Coronary angiography findings were consistent with a critical stenosis in the left circumflex artery

**Fig. 5:**
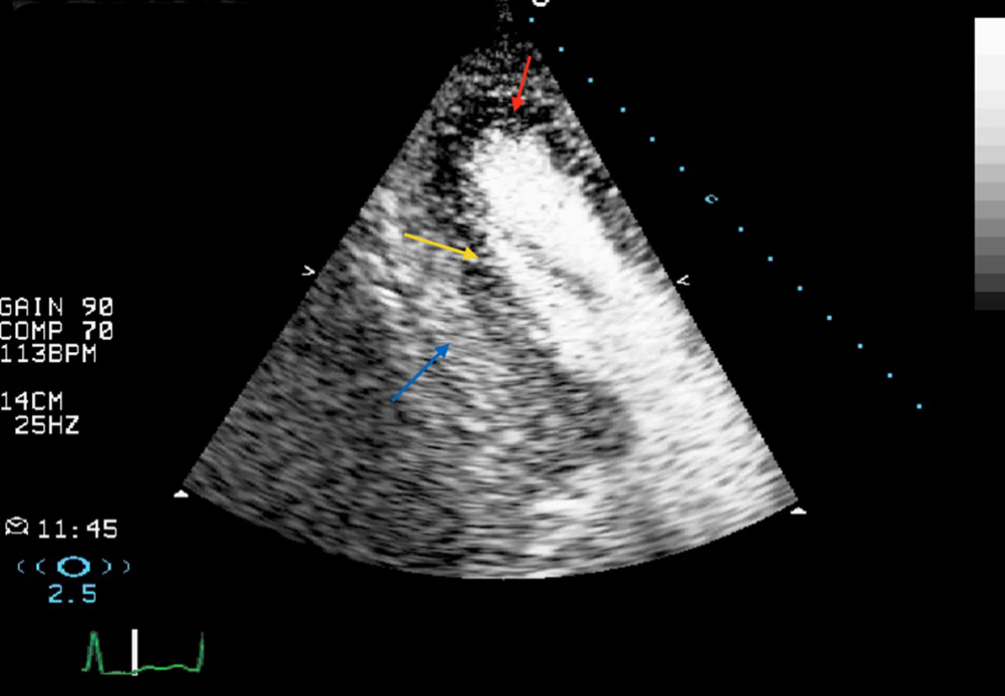
Another case of real-time low-MI myocardial contrast echocardiography that demonstrates a myocardial perfusion defect in an apical two-chamber view. *Blue arrow* epicardial wall, *yellow arrow* subendocardial perfusion defect, *red arrow* transmural perfusion defect. Coronary angiogram showed significant right and left coronary artery disease

*Nondestructive or real-time low-MI imaging techniques *allow for simultaneous assessment of the left ventricular regional wall motion with perfusion imaging. This means a minimum frame rate of 25 MHz is required and thus a lower MI (usually less than 0.2) is used to avoid microbubble destruction, but this is at the cost of a weaker backscatter from the microbubbles. The nondestructive techniques are essentially the same as destructive ones but use a lower amplitude of transmitted waves. In *power modulation *technique (Phillips, USA), multiple pulses are transmitted in each scan line with each alternate phase only in 50% amplitude pulses. The ultrasound scanner doubles the received signals from the 50% amplitude pulses and then subtracts that from the received signal from the 100% amplitude pulses. Therefore, backscattered signals from the myocardial tissue, which produces a linear response, will be cancelled out. Power modulation is considered a highly sensitive technique but has lower resolution and image quality [3]. *Pulse inversion Doppler*, as was explained in high-MI imaging, can also be used in the real-time low-MI MCE. In this technique, essentially, nonlinear scatters from the microbubbles will be detected while linear response from myocardial tissue will be canceled resulting in high-resolution images. However, because it can only receive even harmonics, there will be attenuation artifact, particularly in the basal myocardial segments. The *contrast pulse sequencing *(Siemens, USA*) *is another multiphase technique that uses both alternate amplitude and polarity resulting in high image quality and sensitivity but is still subject to attenuation ([3]; Figs. 4 and 5).

**Fig. 6:**
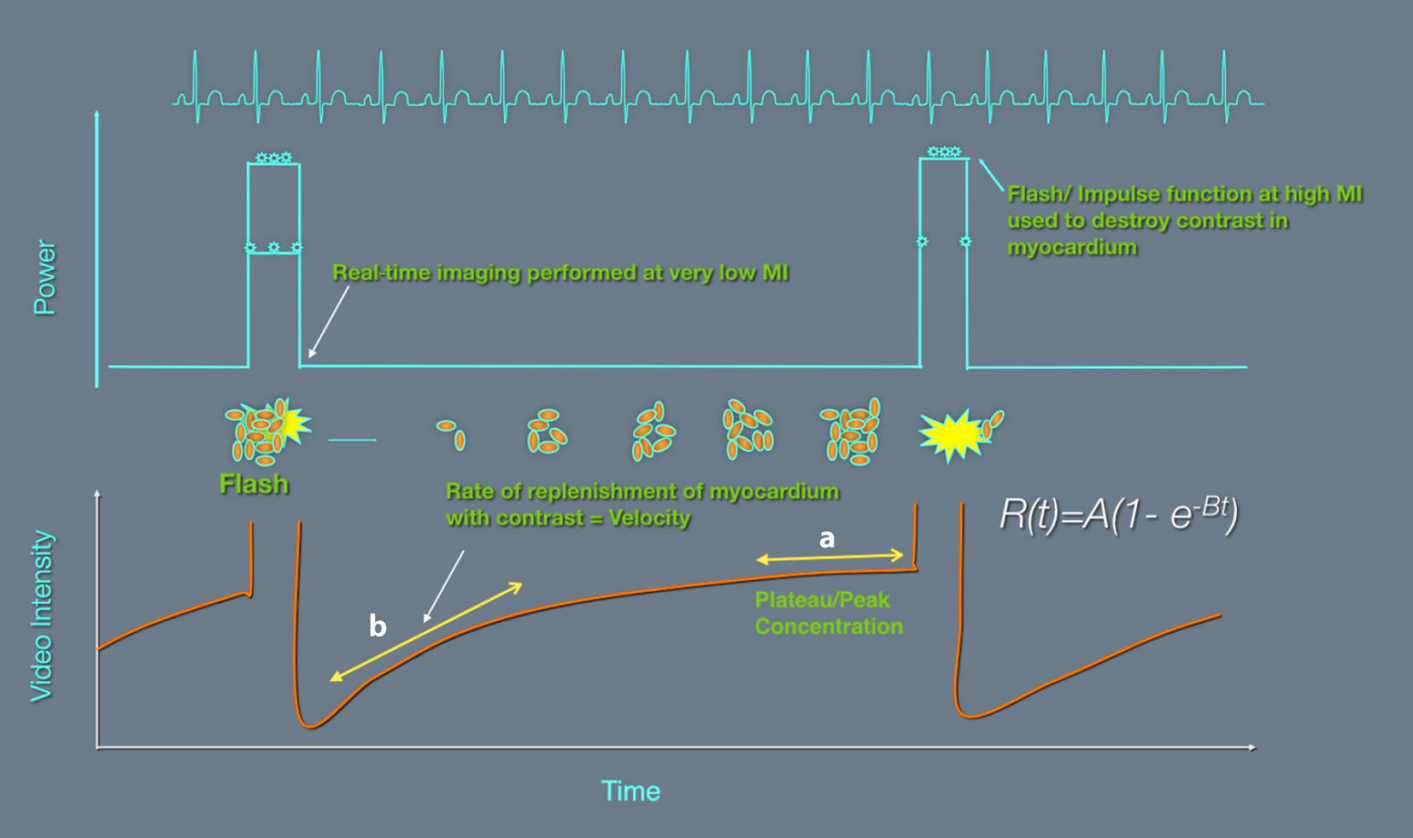
Diagram illustrating the basic principles of quantification of myocardial blood flow by real-time low-MI myocardial contrast echocardiography. The plateau portion of the reperfusion curve is proportional to myocardial blood volume (*A*). The slope of the curve is proportional to blood flow velocity (*B*). R(t) is proportional to myocardial blood flow. (Modified from Monaghan [30])

An important advantage of MCE compared with other noninvasive imaging modalities is the ability to calculate myocardial blood flow. This is measured by multiplying myocardial blood volume and myocardial contrast velocity and was first introduced by Wei and Kaul in 1998, using high-MI intermittent triggered imaging. When the ultrasound pulsing interval is incrementally varied, mean microbubble velocity and peak signal density (representing myocardial blood volume) and thus myocardial blood flow can be measured [23]. Use of the flash option, in real-time low-MI imaging, destroys the UCA in myocardium (while microbubbles remain intact in the left ventricle owing to a higher concentration) and allows for measurement of contrast replenishment velocity and thus quantification of myocardial blood flow (Fig. 6).

A prerequisite for MCE is that the relationship between the contrast concentration and the scatter intensity is linear. With a contrast bolus injection, at a certain level, when the maximum UCA concentration is achieved, the linear relationship is lost. At this point, even a hypoperfused region may appear falsely normal. It is only during the contrast decay that the myocardial contrast intensity and hence the myocardial blood volume can be assessed. Thus, with the bolus injection, maintaining a window where the UCA can be detected and yet not saturated is crucial. Whereas with the contrast infusion, an optimal level of microbubble concentration within the myocardium can be easily achieved. Moreover, since the mean transit time of the microbubbles during contrast injection cannot be calculated, it is impossible to measure myocardial blood flow. For these reasons a contrast infusion is preferred for performing MCE.

#### Stress MCE

Exercise, vasodilators, or inotropes can be used for stress MCE. The incorporation of MCE into an exercise stress test is challenging but has been shown to be feasible with improvement of both left ventricular wall assessment and perfusion [23, 24]. It is known that a perfusion defect precedes a wall motion abnormality in the ischemic cascade. MCE has the ability to detect such perfusion defects during dobutamine stress echocardiography. Dolan et al. demonstrated that the presence of a perfusion defect during dobutamine stress echocardiography, even in the absence of any wall motion abnormality, is an independent prognostic factor for death and nonfatal myocardial infarction [25]. Stress MCE has been studied against the gold standard of coronary angiography in 20 trials with different protocols involving 1683 patients, demonstrating sensitivity and specificity of 83% and 80%, respectively [26]. A meta-analysis investigating the accuracy of stress MCE, single-photon emission computed tomography (SPECT), and dobutamine stress echocardiography against coronary angiography as gold standard also showed a higher sensitivity in favor of stress MCE [27]. Despite several favorable studies, in a more recent European multicenter study using coronary angiography as a gold standard, although stress MCE yielded better sensitivity (75%) in detecting coronary stenosis of >70% against SPECT, it was significantly inferior in specificity (52%).

#### MCE for myocardial viability

MCE can be used for the assessment of myocardial tissue viability since an intact tissue will be replenished with contrast because the capillaries and microvasculature will still be intact, whereas an ischemic or fully infarcted territory will show patchy or absent contrast density. In fact, the contrast density has an inverse relationship with myocardial collagen. MCE has been used for evaluation of the myocardial viability in both acute and chronic settings. Ito in 1992 demonstrated that despite a patent artery related to an infarcted zone, up to 25% of patients did not show myocardial opacification in an infarcted area and thus no myocardial viability, a phenomenon that is called “no flow” and was first described by Kolner in 1974. Other studies have shown the independent prognostic impact of assessment of the infarct-related myocardial region (treated with either thrombolysis or percutaneous intervention) on left ventricular remodeling and death [23]. Perfusion imaging with MCE has also been studied in nonacute coronary artery disease. Quantification of myocardial blood flow with MCE has been shown to improve accuracy in detecting viable myocardium when compared with both dobutamine stress echocardiography and SPECT in those with left ventricular dysfunction and coronary artery disease [28]. The overall sensitivity and specificity of perfusion imaging with MCE have been reported as 85% and 70%, respectively [6].

#### MCE in suspected acute coronary syndrome

A study by Gaibazzi et al. showed that a positive stress MCE predicted a 1-year incidence of acute coronary syndrome in those who presented to the emergency department with chest pain and unremarkable ECG changes and negative troponin findings. This suggests a possible role for bedside stress MCE in risk stratification of these patients [29].

## Safety

Despite the initial concern about the safety of UCAs, there is a universal consensus that contrast echocardiography is safe. Although extremely rare, anaphylactic reaction remains a true side effect that has been reported in approximately 1 in 10,000 patients. Use of UCAs in pulmonary hypertension, right-to-left shunt through a patent foramen ovale, and in critically ill patients that were of initial concern have been proved in several studies to be safe [3].

## Conclusion

Contrast echocardiography should be an integral part of a modern echocardiography laboratory and is considered as a quality control marker. LVO has an established role in a wide range of clinical scenarios in rest echocardiography and improves the accuracy of stress echocardiography. Perfusion imaging with MCE, although supported by a large body of evidence, has not passed the regulatory processes in the EU and USA and thus remains an off-label indication and currently limited to expert centers. Beyond daily clinical use, UCAs are currently being tested in trials for molecular imaging and targeted drug delivery and thrombolysis.
